# How to increase the resectability of initially unresectable colorectal liver metastases: A surgical perspective

**DOI:** 10.1002/ags3.12276

**Published:** 2019-07-11

**Authors:** Katsunori Imai, René Adam, Hideo Baba

**Affiliations:** ^1^ Department of Gastroenterological Surgery Graduate School of Life Sciences Kumamoto University Kumamoto Japan; ^2^ Centre Hépato‐Biliaire AP‐HP Hôpital Universitaire Paul Brousse Villejuif France

**Keywords:** ALPPS, colorectal liver metastases, conversion surgery, two‐stage hepatectomy

## Abstract

Although surgical resection is the only treatment of choice that can offer prolonged survival and a chance of cure in patients with colorectal liver metastases (CRLM), nearly 80% of patients are deemed to be unresectable at the time of diagnosis. Considerable efforts have been made to overcome this initial unresectability, including expanding the indication of surgery, the advent of conversion chemotherapy, and development and modification of specific surgical techniques, regulated under multidisciplinary approaches. In terms of specific surgical techniques, portal vein ligation/embolization can increase the volume of future liver remnant and thereby reduce the risk of hepatic insufficiency and death after major hepatectomy. For multiple bilobar CRLM that were traditionally considered unresectable even with preoperative chemotherapy and portal vein embolization, two‐stage hepatectomy was introduced and has been adopted worldwide with acceptable short‐ and long‐term outcomes. Recently, ALPPS (associating liver partition and portal vein ligation for staged hepatectomy) was reported as a novel variant of two‐stage hepatectomy. Although issues regarding safety remain unresolved, rapid future liver remnant hypertrophy and subsequent shorter intervals between the two stages lead to a higher feasibility rate, reaching 98%. In addition, adding radiofrequency ablation and vascular resection and reconstruction techniques can allow expansion of the pool of patients with CRLM who are candidates for liver resection and thus a cure. In this review, we discuss specific techniques that may expand the criteria for resectability in patients with initially unresectable CRLM.

## 1 INTRODUCTION

The liver is the most common site of metastasis from colorectal cancer, due to portal venous drainage from colon to liver. Approximately half of patients with colorectal cancer develop liver metastases at some point during the course of their disease.^1^, ^2^, ^3^, ^4^ Surgical resection remains the only treatment of choice for curative strategy, with a 5‐year survival rate of up to 67% in selected patients.^5^, ^6^, ^7^, ^8^, ^9^, ^10^, ^11^ Nevertheless, only 20%‐30% of patients with colorectal liver metastases (CRLM) are initially considered to be eligible for surgery,^6^, ^12^, ^13^, ^14^ and liver metastases remain the predominant cause of death for colorectal cancer patients.^15^, ^16^ In patients with metastatic colorectal cancer treated with chemotherapy alone, survival beyond 5 years is unusual.^8^, ^17^, ^18^, ^19^, ^20^, ^21^

The development of surgical techniques, the increasing efficacy of modern chemotherapy with or without biological agents, and the emergence of multidisciplinary approaches have allowed patients with conventionally unresectable CRLM to undergo surgery. Given that surgical resection remains the only form of treatment that offers the possibility of prolonged survival, expanding the potentially resectable pool of patients is crucial.

In confronting unresectability, one of the most important issues is that the definition of unresectability differs among institutions. The definition of unresectability is also evolving with the development of surgical techniques and chemotherapy. Nowadays, unresectability should be considered based on both technical and oncological criteria.^22^, ^23^, ^24^ Historically, several tumor factors that represent massive tumor loads such as multinodular tumors, larger tumor size, bilobar distribution, and the presence of extrahepatic disease were used to classify liver metastases as unresectable, because these variables were prognostic factors for survival after hepatectomy for CRLM. However, recent advances in surgical techniques, in combination with modern effective chemotherapy, enable surgeons to overcome these issues, and these factors are no longer an absolute contraindication for surgery. According to European Society of Medical Oncology (ESMO) consensus guideline 2016, patients with CRLM can be categorized into groups on technological and oncological criteria (Table 1). However, because the decision of unresectability will change according to the transition of the time, a precise definition in response to the demands of the times should be required.

**Table 1:** Contraindications to hepatic resection in patients with colorectal liver metastases (European Society of Medical Oncology consensus guideline 2016, Van Custem et al.)

Category	
Technical (A)	
1. Absolute	Impossibility of R0 resection with ≥30% liver remnant
	Presence of unresectable extrahepatic disease
2. Relative	R0 resection possible only with complex procedure (portal vein embolization, two‐stage hepatectomy, hepatectomy combined with ablation)
	R1 resection
Oncological (B)	
1	Concomitant extrahepatic disease (unresectable)
2	Number of lesions ≥ 5
3	Tumor progression

Note

Patients should be categorized as A1 or A2/B1, B2 or B3.

**Figure 1:**
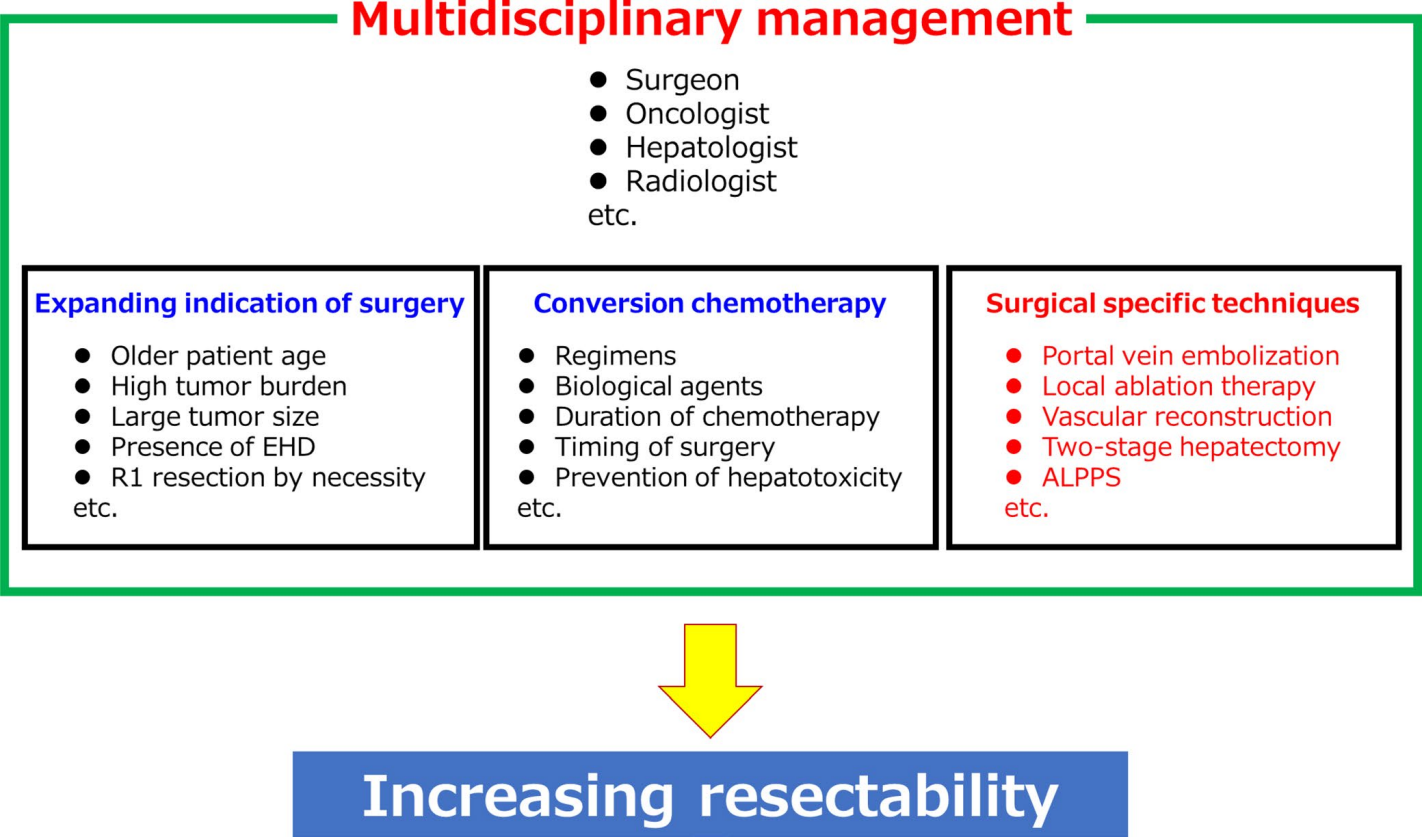
Three material factors for increasing resectability. EHD, extrahepatic disease; ALPPS, associating liver partition and portal vein ligation for staged hepatectomy

How can we increase the resectability of initially unresectable CRLM? We enumerate three material factors for improving resectability (Figure 1): (a) expanding the indication of surgery (eg, for older patients, larger tumor size, and R1 resection by necessity); (b) conversion chemotherapy (eg, considering biological agents and the duration of chemotherapy); and (c) specific surgical techniques (eg, portal vein embolization [PVE], two‐stage hepatectomy [TSH], and associating liver partition and portal vein ligation for staged hepatectomy [ALPPS]); which are regulated under multidisciplinary management (eg, involving surgeons, oncologists, and radiologists).

Among the three, this review discusses the specific surgical techniques that may expand the criteria for resectability in patients with initially unresectable CRLM.

## 2 SPECIFIC TECHNIQUES TO INCREASE RESECTABILITY

### 2.1 PVE

PVE was first described by Makuuchi et al^25^ in the 1980s. This procedure induces atrophy of the embolized liver lobe with compensatory hypertrophy of the non‐embolized contralateral liver lobe, and can thereby reduce the risk of hepatic insufficiency after major hepatectomy in patients with an insufficient future liver remnant (FLR).^26^, ^27^, ^28^ Several studies have reported that PVE has no adverse effect on survival in patients with CRLM who have undergone major hepatectomy.^28^, ^29^, ^30^, ^31^, ^32^

PVE approaches include transileocolic and transhepatic techniques. The transileocolic approach requires a mini‐laparotomy. Although a previous meta‐analysis demonstrated that the major complication rates of transileocolic and transhepatic PVE were comparable,^27^ the transhepatic approach has become standard due to the recent development of radiological intervention techniques. Transhepatic PVE is performed percutaneously, by an ipsilateral or contralateral approach. The advantage of the ipsilateral approach is that it does not require puncture of the FLR; that is, it can reduce the risk of injury of vessels in the FLR. Given that the vessels of the FLR should be carefully preserved for the subsequent planned surgery, the transhepatic ipsilateral approach is recommended if possible, despite its relative complexity.

One of the concerns about preoperative PVE is that stimulation of liver hypertrophy can also accelerate tumor growth in the embolized and non‐embolized liver lobe.^32^, ^33^, ^34^, ^35^, ^36^ Portal flow reduction in the embolized liver leads to an increase of arterial blood flow and, subsequently, growth of tumors including micrometastases could be induced, because liver tumors are mostly supplied by arterial blood. Although tumors in the embolized liver lobe will be removed by surgery, those in the non‐embolized liver lobe could be also stimulated, leading to a cause of unresectability or a risk of early tumor recurrence. One possible way to reduce the risk of unresectability would be chemotherapy after PVE, although, to our knowledge, there are no studies providing evidence of the efficacy of chemotherapy between PVE and hepatectomy. Meanwhile, preoperative PVE should probably be indicated for unilobar disease, and in the case of multiple bilobar diseases, which requires hypertrophy of the FLR, two‐stage surgery may be indicated.

### 2.2 Local ablation therapy in combination with hepatectomy

Local ablation therapy including radiofrequency ablation (RFA) and microwave ablation (MWA) is a thermal ablation technique that is designed to produce localized tumor destruction by heating the tumor and the surrounding liver tissue. Nowadays RFA is adopted worldwide as a safe, effective, well tolerated, and less invasive technique for small liver tumors. However, the therapeutic role of RFA is yet to be decided for CRLM. A randomized CLOCC study (RFA + chemotherapy vs chemotherapy alone) reported that RFA + chemotherapy offered better long‐term survival than chemotherapy alone in patients with unresectable CRLM (8‐year overall survival [OS] rate: 35.9% vs 8.9%, respectively).^37^ We previously reported that RFA in combination with hepatectomy achieved outcomes comparable to hepatectomy alone in a propensity score‐matched setting.^38^ Another study reported that RFA in combination with hepatectomy gave poorer survival than hepatectomy alone; however, in the setting of tumor number ≥ 4, long‐term survival rates were similar between the two groups.^39^ These results suggest that in selected patients and selected tumors, adding RFA to hepatectomy may be justified in the treatment of multiple CRLM.

In essence, we should not support the unconsidered use of RFA in the treatment of CRLM. Surgeons should always strive for resection with a clear margin. However, most patients with CRLM are not candidates for hepatectomy at the time of diagnosis. In these circumstances, adding RFA to hepatectomy would expand the pool of patients who can receive radical treatment.^23^ With this view, RFA should be strictly indicated for appropriate patients and appropriate tumors. We consider appropriate indications to be as follows: (a) unresectable or deeply located small tumors (≤2 cm) within the FLR, which require extended parenchymal resection; and (b) tumors that have responded to preoperative chemotherapy. Tumors that demonstrate no response to chemotherapy have potentially more malignant behavior and a higher local recurrence rate, and RFA should therefore be contraindicated for such tumors.^40^

In addition to its lower invasiveness, the benefit of adding RFA to hepatectomy is that it allows the uninvolved functional liver parenchyma to be preserved. Several recent studies have reported the usefulness of parenchyma‐preserving hepatectomy for CRLM, and Mise and colleagues found that parenchyma‐preserving hepatectomy improved survival in the event of intrahepatic recurrence, possibly due to an increase in the likelihood that the patients would be able to undergo salvage repeat hepatectomy.^41^ In our previous study, salvage hepatectomy for intrahepatic recurrence was more frequently performed in patients who underwent RFA in combination with hepatectomy than in those who underwent hepatectomy alone.^38^ Endeavoring to preserve liver parenchyma should lead to an increase in the number of patients who can undergo salvage hepatectomy for recurrence and in subsequent prolonged survival.

During the past several years MWA has gained acceptance alternative to RFA. Theoretical benefits of MWA over RFA include larger ablation zone, shorter duration, and no heat‐sink effect.^42^ There are some studies reporting the outcomes of MWA for colorectal liver metastases as a safe and effective modality for use in the treatment of CRLM patients.^43^, ^44^, ^45^, ^46^, ^47^, ^48^, ^49^ Correa‐Gallego et al. reported that ablation‐site recurrence of CRLM was lower with MWA compared with RFA (6% vs 20%) in the matched cohort analysis including 254 tumors from 134 patients. For RFA, it is said that there is relationship between higher rate of ablation‐site recurrence and proximity of the tumor to large venous structure because of a heat‐sink effect.^50^ In contrast, MWA may be less affected by the heat‐sink effect, leading to better local control rate. However, van Tilborg et al^47^ reported that MWA has a higher complication rate than RFA for peribiliary CRLM, maybe due to its more aggressive heat production. It is important to use these thermal ablation techniques after understanding each characteristic enough.

### 2.3 Vascular resection and reconstruction

Major vascular invasion is one of the common reasons for unresectability in patients with CRLM. When the tumor is invading either the portal vein bifurcation, all three hepatic veins, or the inferior vena cava (IVC), vascular resection and reconstruction would be required. Removal of a tumor with involvement of these major vessels has been thought to be technically demanding. However, recently, considerable advances in vascular surgery and liver transplantation have made these procedures possible.

For portal vein resection and reconstruction, primary end‐to‐end anastomosis is preferred over the use of grafts if possible.^51^ If primary anastomosis is impossible, several potential autologous conduits are available, including the left renal vein, superficial femoral vein, hepatic vein (from the resected liver), jugular veins, or saphenous vein modified to a spiral graft.

Hepatic vein/IVC resection and reconstruction, with liver resection, is now accepted for most liver tumors including CRLM. Reconstructions of IVC include primary suture under a side‐biting clamp, patch reconstruction, or complete replacement of the vena cava with a synthetic or biological graft.^51^ For reconstruction of the hepatic vein, patch reconstruction, autogenous vein graft (eg, external iliac vein, left renal vein, hepatic vein [from resected liver], jugular veins, or customized saphenous vein), or artificial graft have been used.^52^, ^53^, ^54^, ^55^, ^56^

Because many patients with CRLM have received chemotherapy, some of them suffer from chemotherapy‐induced liver injury, and for such patients the FLR volume may be insufficient due to their impaired liver function. In such a situation, even if the tumor is solitary, or if not all three hepatic veins are involved, hepatic vein reconstruction can sometimes be required. Several recent studies reported the usefulness of vascular resection and reconstruction with hepatectomy for CRLM, with acceptable short‐ and long‐term outcomes.^53^, ^54^, ^55^ A larger‐scale study will be required to confirm its benefit, in terms of safety, acceptability, and impact on long‐term outcomes.

### 2.4 Two‐stage hepatectomy

From 1992, a Paul Brousse team introduced the concept of TSH, based on two sequential procedures to remove multiple bilateral tumors that were impossible to remove by a single hepatectomy and then using the liver regeneration obtained after the first procedure; the work was published in 2000.^57^ During the next decade, TSH was developed in combination with portal vein ligation (PVL)/PVE and effective chemotherapy, achieving a 5‐year survival rate of 32%‐64%. The indication of TSH for CRLM is only bilateral, multinodular diseases which could not be resected by a single hepatectomy, even in combination with other specific techniques such as PVE, local ablation therapy, or vascular reconstruction.^58^

**Figure 2:**
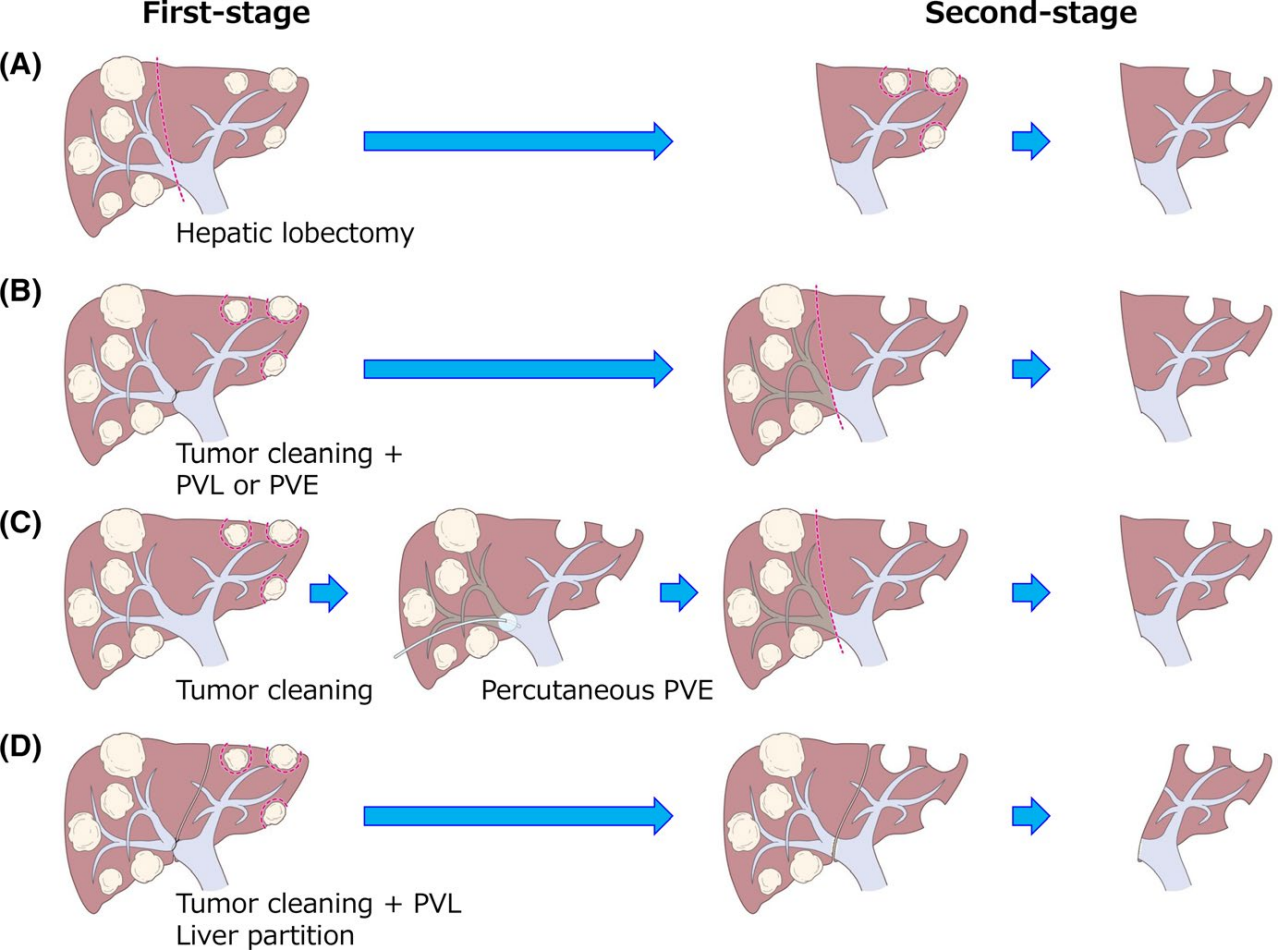
Scheme of staged hepatectomy for colorectal liver metastases. (A) Right‐first approach: most of the invaded hemiliver (usually the right lobe) is resected at the first stage, leading to hypertrophy of the contralateral liver lobe. At the second stage, tumor cleaning of the future liver remnant (FLR) is performed, usually by non‐anatomical partial resection. (B) Left‐first approach with portal vein ligation/embolization (PVL/PVE): The less invaded liver lobe (FLR, usually the left lobe) is cleaned of its metastases in combination with intraoperative PVL/PVE at the first stage. At the second stage, the tumor‐bearing liver lobe (deportalized liver lobe) is anatomically removed. (C) Left‐first approach followed by PVE: percutaneous PVE is performed between the first and second stages. (D) ALPPS: the less invaded liver lobe is cleaned of its metastases in combination with intraoperative PVL/PVE and in situ splitting of the hemiliver at the first stage. At the second stage, usually 7‐14 days later, the tumor‐bearing liver lobe is removed

TSH is usually classified into four types as follows (Figure 2). (A) Right‐first approach: the more invaded hemiliver (usually the right lobe) is resected at the first stage, leading to hypertrophy of the contralateral liver lobe. PVL/PVE is not required. At the second stage, tumor cleaning of the FLR is performed, usually by non‐anatomical partial resection. (B) Left‐first approach with PVL/PVE: the less invaded liver lobe (FLR, usually the left lobe) is cleaned of its metastases in combination with intraoperative PVL/PVE at the first stage. At the second stage, the tumor‐bearing liver lobe (deportalized liver lobe) is anatomically removed. (C) Left‐first approach followed by PVE: percutaneous PVE is performed between the first and second stages. (D) ALPPS: a novel form of conventional TSH. The less invaded liver lobe is cleaned of its metastases in combination with intraoperative PVL/PVE and in situ splitting of the hemiliver at the first stage. At the second stage, usually 7‐14 days later, the tumor‐bearing liver lobe is removed. The details are described in the next section. From the viewpoint that liver hypertrophy after PVL/PVE may accelerate tumor growth,^32^, ^33^, ^34^, ^35^, ^36^ the left‐first approach has now become a mainstream strategy of TSH.

The main drawback of the TSH strategy is the failure to complete both sequential procedures; that is, dropout from the second‐stage hepatectomy. A systematic review demonstrated that the failure rate of TSH ranged 0%‐36% (median, 23%), the main reason for failure being disease progression after first‐stage surgery.^59^ We previously reported a failure rate of 35.2%, and main reason was disease progression after first‐stage surgery (88.6%).^60^ In this study, four independent predictive factors for the failure of TSH were identified, namely tumor progression while on first‐line chemotherapy, number of chemotherapy cycles >12, tumor size >40 mm, and carcinoembryonic antigen at hepatectomy >30 ng/mL. A predictive model for failure of TSH was created based on the logistic model. To complete both sequential procedures in the TSH strategy is crucial for prolonged survival in patients who are planned for TSH, and to achieve that, preventing disease progression after first‐stage surgery is the most important issue.

TSH is now an established strategy for patients with multiple bilobar CRLM, in terms of short‐ and long‐term outcomes. According to our recent review,^58^ postoperative complication rates after the first and second stages are 0%‐37% and 11%‐60%, and mortality rates are 0%‐4% and 0%‐6%, respectively. Regarding long‐term outcome, the 5‐year OS rate after completion of TSH ranged 32%‐64%, with a median survival time of 22‐44 months. In this review, we present analyses comparing the survival between TSH and standard one‐stage hepatectomy performed at Paul Brousse Hospital. Among the patients who underwent liver‐curative surgery (R0 or R1 resection), survival for patients who completed TSH (n = 93) was comparable with that of those who underwent one‐stage hepatectomy (n = 940) (5‐year OS: 41.3% vs 48.0%, median 44.3 vs 56.6 months, *P *=* *.40).^58^ These results suggest that if both of the sequential TSH procedures are completed, a long‐term outcome that is comparable with standard one‐stage hepatectomy can be expected.

Because of their more extensive tumor load, patients who undergo TSH experience an extremely high recurrence rate: the 3‐year disease‐free survival rate after TSH was reported as 6%‐27% (median, 20 months).^61^ In our recent report, 1‐, 3‐, and 5‐year disease‐free survival rates were 28.7%, 12.3%, and 10.5%, respectively, in 93 patients who completed TSH.^62^ Among the 81 patients who were able to undergo potentially curative surgery, disease recurrence was observed in 62 (76.5%) during the study period. After TSH completion, repeat surgery for recurrence was performed in 38 patients (51.4%), and their survival was significantly better than those who could not undergo repeat surgery (5‐year survival rate: 45.8% vs 26.3%, *P *=* *.0041). These findings suggest that repeat surgery for recurrence may be crucial for ensuring long‐term survival, even after very invasive surgery such as TSH.

### 2.5 ALPPS

ALPPS is a novel form of TSH. The first report of 25 cases who underwent the ALPPS procedure in five German centers was published in 2012.^63^ In this paper, rapid growth of the FLR with a median hypertrophy rate of 74% after 9 days and a complete resection rate of 100% were reported. Despite initial concerns about high mortality and morbidity rates, this innovative concept has been adopted by many specialized centers around the world.

The main criticism of ALPPS was its high morbidity and mortality rates. Indeed, the first paper reported morbidity and mortality rates of 68% and 12%, respectively.^63^ An international ALPPS registry was subsequently initiated to collect information from multiple centers worldwide from 2012, and in the first report from this registry (202 patients, including 141 patients with CRLM), the 90‐day mortality rate was 8% and the major complication rate (≥ Clavien‐Dindo IIIa) was 36% in CRLM patients.^64^ After that, with accumulating experience, considerable efforts have been made to improve safety, such as development of technical modifications, stricter patient selection, and a deeper understanding of the procedure. Recent data from the ALPPS registry (486 CRLM patients) revealed that the 90‐day mortality rate was 7% and the major complication rate (≥ Clavien‐Dindo IIIa) was 39%, with a completion rate of both stages of 98%.^65^

Since ALPPS was introduced, many surgeons have reported various modifications of this procedure to improve postoperative short‐ and long‐term outcomes, including anterior‐approach ALPPS (using anterior approach with or without the liver hanging technique),^66^, ^67^ hybrid ALPPS (anterior approach + PVE after the first stage),^68^ partial ALPPS (partial liver partition),^69^ mini‐ALPPS (partial liver partition to a depth not exceeding 3‐5 cm + intraoperative PVE)^70^, ALTPS (tourniquet instead of liver partition),^71^ RALLP (radiofrequency ablation instead of liver partition),^72^ ALPTIPS (partial liver partition until the middle hepatic vein + intraoperative transileocecal PVE),^73^ and modified ALPPS with preservation of portal pedicles (preserving portal pedicles at the transection plane) (Table 2).^74^ In addition, ALPPS modifications using minimally invasive approaches, such as laparoscopic^75^ or robotic ALPPS^76^ have been described. However, the reports of these modifications consist of small case series, and larger‐scale or prospective controlled studies will be needed to establish the real roles of these modifications.

**Table 2:** Several modifications of ALPPS procedure

Procedure	Technical features	Liver transection	Benefits	Scheme	Ref
Anterior‐approach ALPPS	Complete liver transection using anterior approach No liver mobilization	Complete down to IVC	Less adhesion Prevention of tumor dissemination	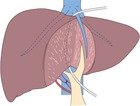	66, 67
Hybrid ALPPS	Complete liver transection using anterior approach No liver mobilization PVE between the stages	Complete down to IVC	Less adhesion Prevention of tumor dissemination	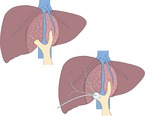	68
Partial ALPPS	Partial liver transection using anterior approach Preserve outflow via the hepatic vein Intraoperative PVE	50%‐80% of the complete transection line	Prevent devascularization of the liver	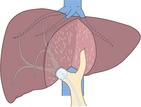	69
Mini‐ALPPS	Partial liver transection using anterior approach No liver mobilization Intraoperative PVE	The depth of liver transection not to exceed 3‐5 cm	Less adhesion Prevention of tumor dissemination Prevent devascularization of the liver	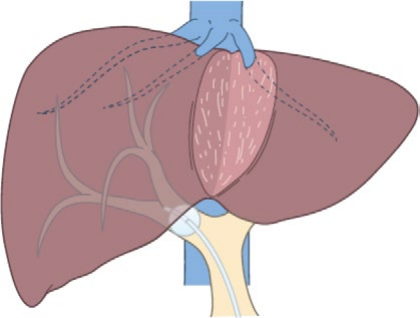	70
ALTPS	Tourniqueting around the transection line and knotted	Just producing a 1‐cm deep groove Tourniquet was placed around the groove and then knotted tightly enough	Less adhesion Less invasiveness at 1st‐stage	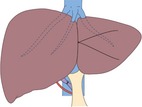	71
RALLPS	Creating an avascular groove at the future transection line	No liver transection	Less adhesion Less invasiveness at 1st‐stage	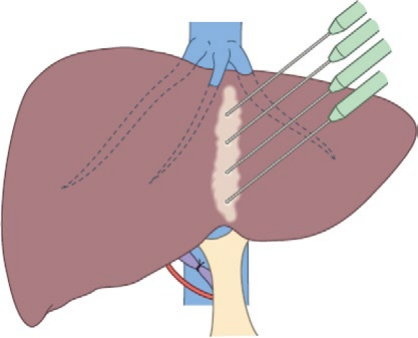	72
ALPTIPS	Partial liver transection using anterior approach Intraoperative PVE	Along the Rex‐Cantlie line until the anterior wall of the MHV is exposed	Less adhesion Prevention of tumor dissemination Prevent devascularization of the liver	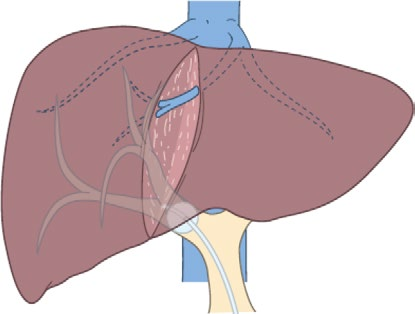	73
Modified ALPPS with preservation of portal pedicles	Preserving portal pedicles during liver transection	Complete but portal pedicles are preserved	Prevent devascularization of the liver	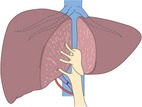	74

Note

ALPPS, Associating liver partition and portal vein ligation for staged hepatectomy; ALTPS, associating liver tourniquet and portal vein occlusion for staged hepatectomy; RALPP, radiofrequency‐assisted liver partition with portal vein ligation; ALPTPS, associating liver partial partition and transileocecal portal vein embolization for staged hepatectomy; PVE, portal vein embolization; IVC, inferior vena cava; MHV, middle hepatic vein.

Compared to conventional TSH, ALPPS has the following advantages: (a) rapid and greater increase of the FLR, (b) shortening of the interval between the two stages, thereby minimizing the dropout risk, (c) maximization of the rate of R0 resections to approaching 100%, and (d) salvage from PVE or PVL failure.^77^ However, the oncological outcomes of ALPPS, relative to TSH, have not been determined. Oldhafer and colleagues reported the risk of early tumor recurrence with disease progression after ALPPS for CRLM.^78^ We previously published a study comparing ALPPS and conventional TSH for CRLM in an early phase of introduction of ALPPS, demonstrating that the OS rate of ALPPS was significantly worse than that of TSH, even in intention‐to‐treat analysis.^79^ However, two other studies reported no difference in OS between ALPPS and TSH.^80^, ^81^ Recently, multicenter randomized controlled trial (LINGO Trial) including 97 patients with CRLM and FLR < 30% (ALPPS, n = 48; TSH, n = 49) reported that resection rate was significantly higher in the ALPPS arm than in the TSH arm (92% vs 57%, *P* < .0001), and there was no difference in terms of complication and 90‐day mortality rates.^82^ Moris et al^83^ conducted a meta‐analysis comparing the operative results and oncological outcomes between ALPPS and conventional TSH in patient with unresectable CRLM. This meta‐analysis included 657 patients (ALPPS, n = 186; TSH, n = 471), and the kinetic growth rate was significantly faster with the ALPPS vs TSH, although there was no difference in final postoperative FLR. TSH had lower overall and major morbidity compared to ALPPS, and OS was comparable following ALPPS vs TSH. In spite of the initial concerns regarding higher morbidity and mortality rates, considerable efforts have made this technically demanding procedure much safer compared to when the ALPPS procedure was introduced.^84^ However, there are many issues left unsolved in ALPPS, such as still high morbidity and mortality rates, early tumor recurrence, possible stimulation of tumor proliferation due to unprecedent liver hypertrophy, and unconfirmed criteria of patient selection. The important thing is that ALPPS and TSH could be complementary in the treatment of multiple bilateral CRLM, and establishment of patient selection criteria will be warranted in further studies.

## 3 OTHER POSSIBLE METHODS FOR IMPROVING PATIENTS’ OUTCOMES

### 3.1 Minimally invasive liver surgery

Laparoscopic liver resection is nowadays adopted worldwide for the treatment of liver tumors including CRLM. Numerous studies have reported the benefits of laparoscopic liver resection compared with the standard open liver resection, such as reduced intraoperative bleeding, a lower morbidity rate, cost‐effectiveness, and shorter in‐hospital stay.^85^, ^86^, ^87^, ^88^, ^89^, ^90^ In addition, laparoscopic liver resection has been reported to provide comparable long‐term oncological outcomes with open liver resection.^88^, ^89^, ^91^ Another possible benefit is that less invasiveness of laparoscopic liver resection may lead to rapid initiation of postoperative chemotherapy.^92^, ^93^, ^94^ In light of this reduced invasiveness without compromising short‐ and long‐term outcomes, laparoscopic liver resection will probably continue to expand in the treatment of CRLM.

Robotic liver surgery is another possible minimally invasive treatment for CRLM. Robotic liver surgery has the potential to overcome some of the limits of laparoscopic surgery, such as limited degrees of motion of the instruments, unstable camera platforms, and two‐dimensional vision.^95^ However, in the consensus opinion during the second international laparoscopic liver forum held in 2014, there was insufficient data to comment on the applicability of robotic liver resection.^96^ Further data accumulation and prospective large‐scale studies will be required.

### 3.2 Liver transplantation

The indication for liver transplantation for CRLM has been restrictive over time because of high recurrence rates and poor outcomes. Given the systemic nature of metastatic disease and need for immunosuppressive therapy after surgery, liver transplantation for CRLM has been controversial. However, recent advances in surgical techniques, advent of more effective systemic chemotherapy, development of imaging modality, and improvements of perioperative management including novel immune‐modulating agents have led to the re‐evaluation of liver transplantation as a treatment option for unresectable CRLM. A recent systematic review reported that estimated cumulative survival rate after liver transplantation was 85.2% (at 1 year), 48% (at 3 years), and 34.6% (at 5 years), respectively, and disease‐free survival rate at 1 year was 38.9%.^97^ Due to the small sample size and lack of studies comparing liver transplantation with current standard of care, it is difficult to evaluate its applicability as a treatment of choice in patients with unresectable CRLM. Now some clinical trials are ongoing and will provide high‐quality evidence regarding the role of liver transplantation in the management of CRLM.

## 4 CONCLUSIONS

For patients with CRLM, surgical resection is the only treatment of choice for prolonged survival and a chance of cure. Given this, how to increase resectability is one of the most crucial outstanding issues. Because we currently have various surgical options to improve resectability, we should use all available techniques to explore the possibilities of surgical resection, working in a multidisciplinary manner with specialists in hepatobiliary, colorectal, and thoracic surgery, and across hepatology, oncology, and radiology.

## DISCLOSURE

Funding: There was no funding for the present study.

Conflict of interest: The authors declare they have no conflict of interest.
